# Structural insights into substrate recognition by the type VII secretion system

**DOI:** 10.1007/s13238-019-00671-z

**Published:** 2019-11-23

**Authors:** Shuhui Wang, Kaixuan Zhou, Xiaolin Yang, Bing Zhang, Yao Zhao, Yu Xiao, Xiuna Yang, Haitao Yang, Luke W. Guddat, Jun Li, Zihe Rao

**Affiliations:** 1 Shanghai Institute for Advanced Immunochemical Studies and School of Life Science and Technology, ShanghaiTech University, Shanghai, 201210 China; 2 CAS Center for Excellence in Molecular Cell Science, Shanghai Institute of Biochemistry and Cell Biology, Chinese Academy of Sciences, Shanghai, 200031 China; 3 State Key Laboratory of Medicinal Chemical Biology, College of Life Sciences and College of Pharmacy, Nankai University, Tianjin, 300353 China; 4 Laboratory of Structural Biology, Tsinghua University, Beijing, 100084 China; 5 National Laboratory of Biomacromolecules, CAS Center for Excellence in Biomacromolecules, Institute of Biophysics, Chinese Academy of Sciences, Beijing, 100101 China; 6 School of Chemistry and Molecular Biosciences, The University of Queensland, Brisbane, QLD 4072 Australia; 7 University of Chinese Academy of Sciences, Beijing, 100101 China

**Keywords:** type VII secretion system, *Mycobacterium tuberculosis*, ATPase, virulence factor, substrate recognition

## Abstract

Type VII secretion systems (T7SSs) are found in many disease related bacteria including *Mycobacterium tuberculosis* (*Mtb*). ESX-1 [early secreted antigen 6 kilodaltons (ESAT-6) system 1] is one of the five subtypes (ESX-1~5) of T7SSs in *Mtb*, where it delivers virulence factors into host macrophages during infection. However, little is known about the molecular details as to how this occurs. Here, we provide high-resolution crystal structures of the C-terminal ATPase_3_ domains of EccC subunits from four different *Mtb* T7SS subtypes. These structures adopt a classic RecA-like **ɑ**/**β** fold with a conserved Mg-ATP binding site. The structure of EccCb1 in complex with the C-terminal peptide of EsxB identifies the location of substrate recognition site and shows how the specific signaling module “LxxxMxF” for *Mtb* ESX-1 binds to this site resulting in a translation of the bulge loop. A comparison of all the ATPase_3_ structures shows there are significant differences in the shape and composition of the signal recognition pockets across the family, suggesting that distinct signaling sequences of substrates are required to be specifically recognized by different T7SSs. A hexameric model of the EccC-ATPase_3_ is proposed and shows the recognition pocket is located near the central substrate translocation channel. The diameter of the channel is ~25-Å, with a size that would allow helix-bundle shaped substrate proteins to bind and pass through. Thus, our work provides new molecular insights into substrate recognition for *Mtb* T7SS subtypes and also a possible transportation mechanism for substrate and/or virulence factor secretion.

**Electronic supplementary material:**

The online version of this article (10.1007/s13238-019-00671-z) contains supplementary material, which is available to authorized users.

## Introduction

*Mycobacterium tuberculosis* (*Mtb*), the pathogen of human tuberculosis (TB), infects one-third of the world’s population resulting in more than one million deaths annually (World Health Organization 2018). Whilst there are several drugs available to treat TB, these are required to be administered over a long time-frames (up to years or longer), can be expensive and can have serious side-effects. Moreover, these current medications are losing their effectiveness due to the emergence of extensively and multi-drug resistant strains of *Mtb*. Thus, there is an urgent call for new anti-TB therapies to be developed.

One potential drug target for *Mtb* is the type VII secretion system (T7SS), which secretes virulence factors during host infection (Stanley et al. 2003). However, very little is known about its molecular features to use as a target for rational structure-based drug design. *Mtb* T7SS was first identified by comparing the genome of *Mtb* H37Rv strain with those of the attenuated vaccine strains of *Mycobacterium bovis* Bacille Calmette-Guérin (BCG) and *Mycobacterium microti* (Pym et al. 2002). A 9.5 kb genomic segment, named RD1, was deleted from all vaccine strains (Mahairas et al. 1996; Behr et al. 1999). The RD1 region encodes nine proteins including two virulence factors, the 6 kDa early secretory antigenic target ESAT-6 (also called EsxA) (Sorensen et al. 1995; Brodin et al. 2004) and its dimerization partner, culture filtrate protein CFP-10 (also known as EsxB) (Berthet et al. 1998), which together form a newly identified and highly specialized secretion pathway, the ESX-1 secretion system [early secreted antigen 6 kDa (ESAT-6) system 1] (Stanley et al. 2003). In the genome of *Mtb*, five paralog esx gene clusters (ESX-1 to -5) have now been identified as T7SSs (Bitter et al. 2009). Each esx cluster encodes small secreted proteins including the Esx proteins themselves, a cytosolic ATPase EccA, and the core transmembrane components EccB, EccC, EccD, EccE and MycP (Bitter et al. 2009). The small secreted proteins are about 100 amino acids in length, containing the conserved WxG and Yxxx[D/E] motifs (Daleke et al. 2012).

The T7SSs play several important roles in *Mtb*. The ESX-1 secretion system is the best studied T7SS amongst the five ESXs. It secretes not only the major virulence factors EsxA and EsxB, but also ESX-1 secretion-associated proteins, EspA, EspB and EspC (Brodin et al. 2006; McLaughlin et al. 2007; Raghavan et al. 2008; Carlsson et al. 2009; Champion et al. 2009). Single-cell fluorescence resonance energy transfer (FRET) observed a pore-forming toxin formed by the ESX-1 secreted EsxA (Smith et al. 2008), finally allowing *Mtb* escape from the innate host immune responses (McLaughlin et al. 2007; van der Wel 2007; Wong and Jacobs 2011; Simeone et al. 2012). ESX-1 can also regulate host immunoreaction and contribute to granuloma formation and bacterial dissemination (Koo et al. 2008; Davis and Ramakrishnan 2009; Carlsson et al. 2010; Volkman et al. 2010; Stoop et al. 2011). Interestingly, ESX-1 is also found to be essential for conjugal DNA transfer in some mycobacteria (Flint et al. 2004; Gray et al. 2013; Derbyshire and Gray 2014). The mycobacterial ESX-3 secretion system has a function in metal homeostasis mediated by mycobactin (Siegrist et al. 2009; Serafini et al. 2013). The EsxG-EsxH complex, which is the substrate for ESX-3, is suggested to strongly induce interferon gamma secretion in T cells of mice infected with *Mtb* (Skjøt et al. 2002; Majlessi et al. 2003; Hervas-Stubbs et al. 2006). ESX-5 is the most recently evolved ESX system which is only present in slow-growing mycobacteria (Gey Van Pittius et al. 2001), and is the major pathway for secretion of PE family (Pro-Glu motif containing) and PPE family (Pro-Pro-Glu motif containing) proteins which are localized at the mycobacterial cell surface (Sampson 2011). *Mycobacteriummarinum esx-5* mutant showed hyper virulence in adult zebrafish, suggesting ESX-5 might have a role in downregulation of the host immune response (Weerdenburg et al. 2012). ESX-2 and ESX-4 may be non-essential systems and not host-oriented (Cole et al. 2001; Singh et al. 2015). Nevertheless, ESX-4 was reported to be crucial for conjugal recipient activity in *Mycobacteriumsmegmatis* (Gray et al. 2016).

**Figure 1:**
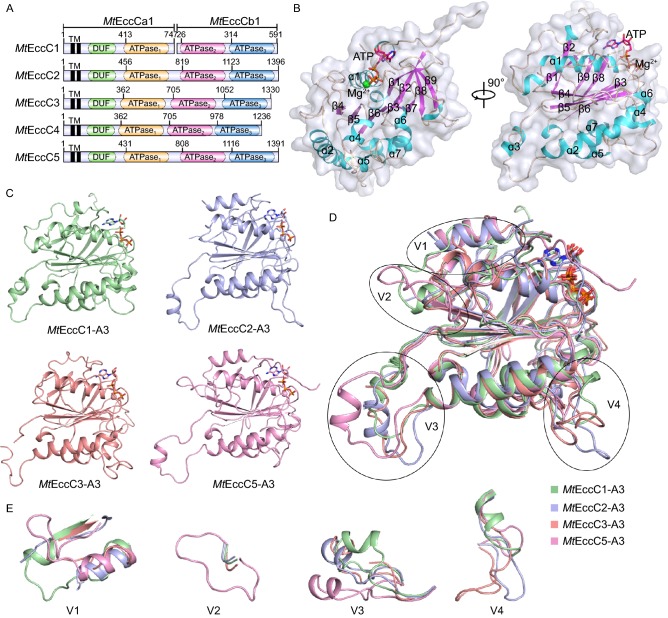
**Overall structure of*Mt*EccC-ATPase**_**3**_. (A) Domain arrangements of EccC proteins from *Mycobacterium tuberculosis*. (B) Cartoon and surface representation for *Mt*EccCb1-ATPase_3_. The helices and beta-sheets are colored cyan and magenta, respectively. ATP and the magnesium ion, are shown in stick representation and as a sphere, respectively. (C) Cartoon representation of *Mt*EccCb1-ATPase_3_ (C1A3, pale green), *Mt*EccC2-ATPase_3_ (C2A3, light blue), *Mt*EccC3-ATPase_3_ (C3A3, salmon) and *Mt*EccC5-ATPase_3_ (C5A3, pink). (D) Superposition of the four structures in (C). Structures of the four variable regions (V1–V4) are marked with circles. (E) Close-up views of the four variable regions in (D)

Using negative staining electron microscopy, the core ESX-5 apparatus from *Mycobacteriumxenopi*, which consists of EccB5, EccC5, EccD5 and EccE5, has been shown to form a 1.8 MDa membrane complex with six-fold symmetry (Beckham et al. 2017). EccC, the motor subunit of T7SS, is essential to assemble a stable membrane complex for the secretion process (Houben et al. 2012). It contains a two-pass transmembrane domain, an unknown function domain (DUF), and three Fts-SpoIIIE-like ATPase domains (ATPase_1_, ATPase_2_, and ATPase_3_) (Pallen 2002). For ESX-1 in *Mtb*, EccC is split between the ATPase_1_ and ATPase_2_ domains, into two proteins, named EccCa1 and EccCb1 (Fig. 1A). Yeast two-hybrid experiment showed that the seven residues at the C-terminus of EsxB interact with EccCb1 as a signal sequence required for secretion (Champion et al. 2006). The flexible C-terminus of PE25, a substrate of ESX-5, has also been shown to be important for secretion (Daleke et al. 2012). In addition, a highly conserved motif (Yxxx[D/E]) has been identified in all known mycobacterial T7SS substrates, and is absolutely required for secretion (Daleke et al. 2012). The structure of the cytoplasmic portion of EccC in complex with the signal sequence of EsxB from *Thermomonospora curvata* and an experiment where the C-terminal sequences between *Tc*EsxB and *Mt*EsxB were swapped indicates that EccC specifically recognizes substrates from different species (Rosenberg et al. 2015). Thus, though there is some data regarding substrate recognition by T7SS, the molecular details about how this works have remained limited, especially in *Mtb*.

Here we have determined the crystal structures of the EccC-ATPase_3_ domains of ESX-1, ESX-2, ESX-3 and ESX-5 from *Mtb* in a pre-activated state. Amongst them, we observed ATP binds in a similar mode at a highly conserved nucleotide-binding site. The structure of EccCb1 in complex with EsxB shows the precise interactions between the ATPase_3_ domain and the specific signaling motif (LxxxMxF) at the C-terminus of EsxB after a translation of the bulge loop. Sequence and structural comparisons reveal that the substrate recognition pockets for the distinct *Mtb* T7SS subtypes differ significantly. These findings provide new insights into substrate recognition by T7SS, thus broadening our knowledge as to how T7SS secretes substrates and virulence factors.

## Results

### Overall structure of EccC-ATPase_3_

An initial attempt to obtain the crystal structure of EccC fragment from *Mtb* containing three or two ATPase domains was failed. Due to the significance of ATPase_3_ domain in substrate interaction and recognition, we then tried to focus study on this region. Firstly, we solved the structure of the ATPase_3_ domain of EccCb1 from *Mtb* at 2.10 Å resolution. The data collection and refinement statistics for this structure are given in Table S2. EccCb1-ATPase_3_ crystallized with two molecules in the asymmetric unit. The overall structure adopts a classic RecA-like ɑ/β fold, with a central five-stranded (β7-β3-β6-β5-β4) parallel β-sheet, which is further extended by an additional anti-parallel sheet that includes β2-β1-β9-β8. The combined sheet wraps around helix ɑ1 on one side while ɑ2 and other four short helices (ɑ4, ɑ5, ɑ6 and ɑ7) cover the other side (Fig. 1B). The nucleotide-binding site is located at the N-terminal end of helix ɑ1. A fragment containing ɑ3 protrudes out of the main body of the structure from the end of ɑ2. It then loops back to connect to β5.

To explain structure-function differences amongst EccC-ATPase_3_ domains, the crystal structures of *Mtb* ATPase_3_ from ESX-2, -3, -5 were determined at 2.20 Å, 1.97 Å and 2.00 Å resolution, respectively (Fig. 1C and Table S2). Though the sequence identities are only 20%~30% amongst the four ATPase_3_ domains, their atomic structures can be superimposed (Fig. 1D) and have *r*.*m*.*s*.*d*. values in the range 0.99~2.02 Å by comparing Cɑ atom pairs (Table S3). However, four structurally variable regions were observed (Figs. 1E and S1). One is at the region following the N-linker which connects to the ATPase_2_ domain (variable region 1, V1). This region may be related to interactions with ATPase_2_ domain and domain movement during substrate translocation. The second is the loop connecting ɑ1 and β4 (V2), which is extremely long in EccC5-ATPase_3_. V3 is located at the protruding segment between ɑ2 and β5 and has different orientations in all of the structures. This region is possibly responsible for hexamer assembly (see DISCUSSION). V4 is the region located between β5 and ɑ5 and has a variable length across the different ATPase_3_ domains. Due to its gating position at the potential central channel of the hexamer model, we speculate that this region may be related to specific interactions with secreted substrates.

A structure-based Dali search (Holm and Laakso 2016) using the model of EccCb1-ATPase_3_ showed a homologous EccC-ATPase_3_ structure of T7SS from *Thermomonospora curvata* (PDB code: 4NH0) (Rosenberg et al. 2015) as the top-hit with an *r*.*m*.*s*.*d*. of 1.7 Å. The V1 and V4 regions can also be superimposed in the two structures. However, *Tc*EccC-ATPase_3_ has an additional β-strand instead of a bulge loop near β4. VirB4 (PDB code: 4AG6) (Walldén et al. 2012) and motor domain of FtsK (PDB code: 2IUT) (Massey et al. 2006) are also observed to have similar structures to EccCb1-ATPase_3_. The former is the energetic ATPase in the Type IV secretion system, which implies that our ATPase domain structures have a similar energetic function during protein secretion. The latter is a DNA translocase that coordinates chromosome segregation and cell division in bacteria. A functional hexameric model for substrate translocation in that structure could be used as a reference model for EccC-ATPase_3_ (see DISCUSSION).

### The highly conserved nucleotide-binding site

**Figure 2:**
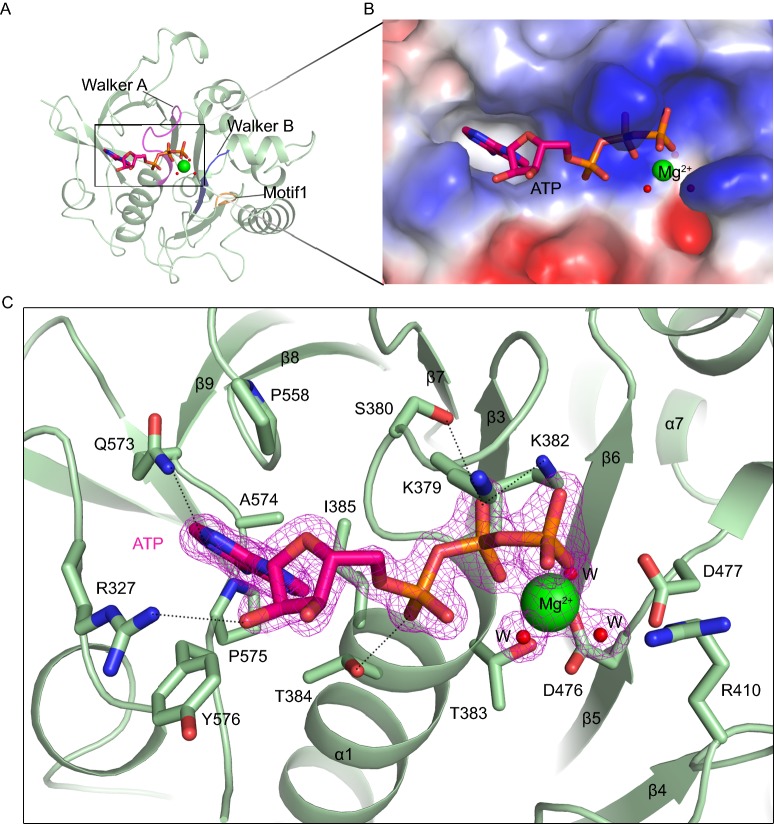
**The nucleotide-binding site of*Mt*EccC-ATPase**_**3**_. (A) The location of the nucleotide-binding site in *Mt*EccCb1-ATPase_3_. ATP and the magnesium ion, are shown as stick representations and as a sphere, respectively. The Walker A, Walker B and Motif 1 are colored magenta, blue and orange, respectively. (B) Close-up view of the nucleotide-binding pocket shown in electrostatic surface representation. ATP and the magnesium ion, are shown as stick representations and as a sphere, respectively. (C) Interactions between Mg^2+^, ATP and nearby amino acid residues. Green and red spheres represent Mg^2+^ and water molecules, respectively. The 2*F*_o_-*F*_c_ density map (pink mesh) of ATP, Mg^2+^ and the coordinated water molecules is contoured at 1 σ. The dashed lines represent hydrogen bonds between ligands and the protein. W, water molecule

It has been demonstrated that ATP binding to the ATPase_3_ of EccC is essential for secretion (Rosenberg et al. 2015). In our structures, ATP and a magnesium ion bind to the nucleotide-binding site in a similar manner (Fig. S2A). This site is bordered by the clearly recognizable Walker A (GXXXXG[K/R][T/S]) and Walker B (hhhhDD with h = hydrophobic residue) motifs which include residues 376–383 and residues 472–477, respectively in *Mt*EccCb1-ATPase_3_ (Figs. 2A and S1). In addition, we found a motif, Motif1 (Dx[R/K]), that also participates in nucleotide binding. For EccCb1-ATPase_3_, ATP-Mg^2+^ is inserted into a deep pocket (Fig. 2B). The adenosine ring of ATP is clamped in place by Arg327, Pro558, and Tyr576 and surrounded by Thr384, Ile385, Gln573, Ala574 and Pro575 (Fig. 2C). The ribose moiety of ATP is more solvent exposed but one of the ribose hydroxyl groups does form a hydrogen bond with the side-chain of R327. The tri-phosphate group is held in the groove at the N-terminus of helix ɑ1 mainly through main-chain interactions. The side chain of the conserved Lys382 in the Walker A motif points to the region between the β- and γ-phosphates implying that it may play an important role in ATP hydrolysis. Other positively charged residues, such as Lys379 in Walker A and Arg410 in Motif1, are also observed near the phosphate groups in the binding site and may also be involved in ATP tethering during hydrolysis.

The β- and γ-phosphates and Thr383, together with three water molecules are ligands for the Mg^2+^, and they form a regular octahedron around this ion (Fig. 2C). The six Mg-O distances are within the expected range of 2.0~2.2 Å. Interestingly, the two Asp residues (Asp476 and Asp477) in the Walker B motif, which usually contribute to stabilization of the Mg^2+^ ion, are too far away to interact directly with the Mg^2+^ ion in our model. Instead, they mediate stability indirectly through interacting with water molecules coordinated to the metal ion (Fig. 2C). At this point, EccC-ATPase_3_ may represent a pre-activated state unfavorable for ATP hydrolysis, and this could explain why intact ATP is observed in our structure. Accordingly, little enzymatic activity (the reaction rate is very low) was observed when these ATPase_3_ domains were assayed (Fig. S5). Even the EccC fragment containing two or three ATPase domains has low reaction rate, though the activity of three-domain group was relatively higher than the others. This pre-activated state is similar to the state of ATPase_3_ domain of EccC from *Thermomonospora curvata* (Rosenberg et al. 2015). The conformation of our structure, together with previous reported ATPase_3_ domain of *Tc*EccC, is similar to the inhibited-state of F_1_-ATPase where the inert ATP analog, AMP-PNP is bound (PDB code: 2CK3) (Bowler et al. 2006; Rosenberg et al. 2015). The homologous ATPase_2_ and ATPase_3_ domains of EssC from *Geobacillus thermodenitrificans* also have similar ATPase structures, and also bind ATP (Fig. S2B) (Zoltner et al. 2016). A ConSurf analysis (Ashkenazy et al. 2010; Celniker et al. 2013) of the ATPase_3_ domains of 143 unique EccC proteins from different species and subtypes showed that nucleotide-binding site is highly conserved (Fig. S4A). Previous reported structure of ATPase_1_ domain of *Tc*EccC showed that the nucleotide binding residues were strikingly different from other two domains and there is only a sulfate ion in the active site when co-crystallization with high concentration of ATP (Rosenberg et al. 2015).

### Substrate recognition by *Mt*EccCb1-ATPase_3_

**Figure 3:**
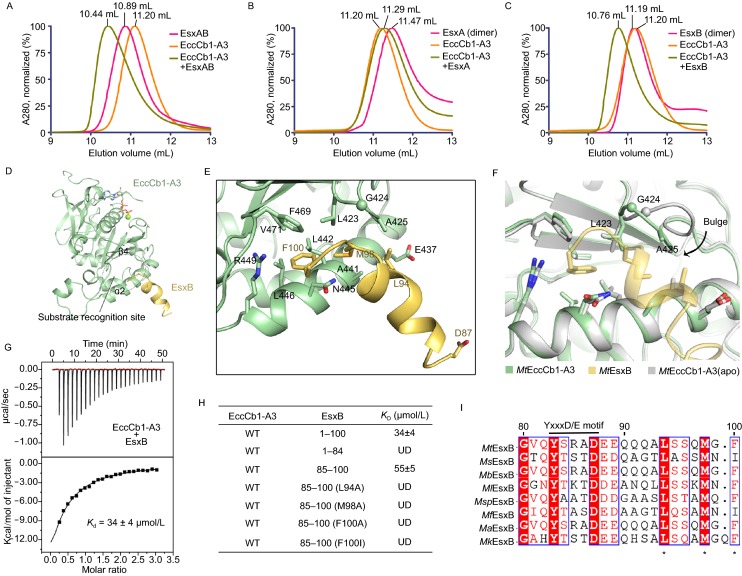
**The C-terminal peptide of*Mt*EsxB interacts with*Mt*EccCb1-ATPase**_**3**_. (A–C) Gel filtration analysis of interactions between Esx proteins and *Mt*EccCb1-ATPase_3_ performed on a Superdex 75 column. The peak volumes are indicated on the top. (A) *Mt*EsxAB binds to *Mt*EccCb1-ATPase_3_ inducing a shift in elution volume. (B) *Mt*EsxA alone does not bind to *Mt*EccCb1-ATPase_3_, thus there is no change in elution volume. (C) *Mt*EsxB binds to *Mt*EccCb1-ATPase_3_, inducing a similar shift in elution volume as observed in (A). (D) Overall structure of *Mt*EccCb1-ATPase_3_ (palegreen) complexed with *Mt*EsxB (yelloworange). The substrate binding site is marked with a dashed circle. (E) Detailed interactions between *Mt*EsxB and *Mt*EccCb1-ATPase_3_. Interacting residues and Asp87 in Yxxx[D/E] motif are shown as sticks. (F) *Mt*EccCb1-ATPase_3_ in complex with *Mt*EsxB is superimposed onto *Mt*EccCb1-ATPase_3_ (*apo*). The bulge in the loop moves closer to *Mt*EsxB to enhance substrate binding. (G) ITC assay shows the binding affinity of *Mt*EsxB to *Mt*EccCb1-ATPase_3_. The data were representative of at least three repetitions. (H) The dissociation constant, *K*_d_, is based on the ITC studies of *Mt*EsxB and its truncations or peptides, to *Mt*EccCb1-ATPase_3_. The data were representative of at least three repetitions. WT, wild type; UD, data was undetermined. (I) Sequence alignment of EsxB from different *Mycobacterium* species including *Mycobacterium tuberculosis* (*Mt*), *Mycobacterium smegmatis* (*Ms*), *Mycobacterium bovis* (*Mb*), *Mycobacterium leprae* (*Ml*), *Mycobacterium sp*. (*Msp*), *Mycobacterium flavescens* (*Mf*), *Mycobacterium africanum* (*Ma*) and *Mycobacterium kyorinense* (*Mk*). The recognition residues are marked with stars. The Yxxx[D/E] motif is also labeled on top of sequences

To understand substrate recognition within ESX-1, interactions between *Mt*EsxA/B and *Mt*EccCb1-ATPase_3_ were investigated. Size exclusion chromatography (SEC) showed that *Mt*EsxA by itself is not able to bind to *Mt*EccCb1-ATPase_3_ (Fig. 3B), however, both the *Mt*EsxAB heterodimer and *Mt*EsxB homodimer do bind to it (Fig. 3A and 3C) with binding affinity constants (*K*_d_ values) of 47 μmol/L and 34 μmol/L, respectively, measured by the isothermal titration calorimetry (ITC) method (Figs. 3G, S6A and S6B). This is consistent with previous reported analysis from homologous proteins from *Thermomonospora curvata* (Rosenberg et al. 2015).

To determine how *Mt*EsxB is recognized by *Mt*EccCb1-ATPase_3_, a structure of the complex was determined at 1.98 Å resolution. However, the main body of the helix bundle of *Mt*EsxB was not observed in the structure. SDS-PAGE analysis of crystal sample showed that *Mt*EsxB degraded severely (Fig. S7A). Only the C-terminal 14 residues of *Mt*EsxB was stable through strongly binding to *Mt*EccCb1-ATPase_3_ according to the high-resolution density. The first 11 residues form an α-helix while the last three residues form a short loop (Figs. 3D and S7B). PISA (Krissinel and Henrick 2007) analysis showed that in total, 414 Å^2^ of surface area is buried at the interacting interface. Most importantly, it is the side chains of Met98 and Phe100 in *Mt*EsxB that insert into the recognition pocket of ATPase_3_, which is located in the gap between ɑ2 and β4. The phenyl ring of Phe100 is sandwiched by Asn445 and Phe469, with Leu442, Leu446, Arg449 and Val471 all located within van der Waals distance (Fig. 3E). Met98 is surrounded by Leu423, Ala425 and Leu442. Additionally, Leu94 is close to ɑ2 and interacts with Glu437 and Ala441. This Leu94 appears to provide extra binding affinity for the complex though it is not inserted into the pocket. A previous report showed that Asp87 in the conserved Yxxx[D/E] motif of *Mt*EsxB is required for secretion (Daleke et al. 2012), however this residue makes no interactions with *Mt*EccCb1-ATPase_3_ in our structure (Fig. 3E). Possibly, an additional component, such as a chaperonin, might interact with this motif to contribute to secretion, or a second recognition by this motif is required in the later secretion steps. *Mt*EccCb1-ATPase_3_ has a unique short bulge loop ^423^LGAGA^427^ near β4, instead of a β-strand observed in other solved *Mt*EccC-ATPase_3_ structures. Superposition of the *Mt*EsxB bound and free *Mt*EccCb1-ATPase_3_ structures showed that the bulge loop moves by ~3.5 Å to allow Gly424 and Ala425 to form interactions with *Mt*EsxB (Fig. 3F). Meanwhile, the side chain of Leu423 also re-orients to be closer to Met98 of *Mt*EsxB. Thus, the shift of bulge loop could further strengthen substrate binding.

To further verify which residues on *Mt*EsxB are crucial for substrate recognition, we made the *Mt*EsxB truncation mutant lacking the 16-residues signal sequence and showed that it has no detectable binding affinity (Figs. 3H and S6C), this is consistent with our structure that showed only C-terminal signaling sequence of *Mt*EsxB was visible. We then synthesized individual peptides corresponding to the C-terminal 16-residues of *Mt*EsxB with site-mutations and then measured their binding affinities with *Mt*EccCb1-ATPase_3_. The *K*_d_ value of the wild-type peptide is 55 μmol/L (Figs. 3H and S6D), a value comparable to the *Mt*EsxB protein. When the side chains of Met98 and Phe100 were individually mutated to alanine, binding is lost completely (Figs. 3H, S6F and S6G). Note that mutation of Leu94 to alanine also resulted in a loss of binding (Figs. 3H and S6E), confirming the role of Leu94. These results were also consistent with the two-hybrid analysis between the two proteins (Champion et al. 2006). Therefore, Leu94, Met98 and Phe100 of *Mt*EsxB are all necessary for substrate recognition. A previous report showed that the G99A mutant abolished substrate binding (Champion et al. 2006; Rosenberg et al. 2015). By referring to our structural information, this could be explained by the loss of flexibility in main chain conformation caused by the addition of the side chain Cβ atom, which prevents Phe100 or Met98 from having an ideal orientation to bind to *Mt*EccCb1-ATPase_3_.

Sequence alignment analysis of EsxB from several *Mycobacterium* species showed that the Leu94 and Met98 are highly conserved, while the C-terminal residue be either Phe or Ile (Fig. 3I). The F100I mutant of *Mt*EsxB peptide was also synthesized but it did not bind to *Mt*EccCb1-ATPase_3_ (Figs. 3H and S6H), suggesting that ESX-1 systems from different *Mycobacterium* species recognize substrates specifically, using either the LxxxMxF or LxxxMxI pattern. Taking this data and analysis together, we have provided detailed structural information on the interactions between *Mt*EccCb1-ATPase_3_ and *Mt*EsxB, which gives a clear explanation for the substrate recognition pattern at the C-terminus of EsxB for *Mtb* ESX-1.

### Structural comparison for substrate-specific recognition

**Figure 4:**
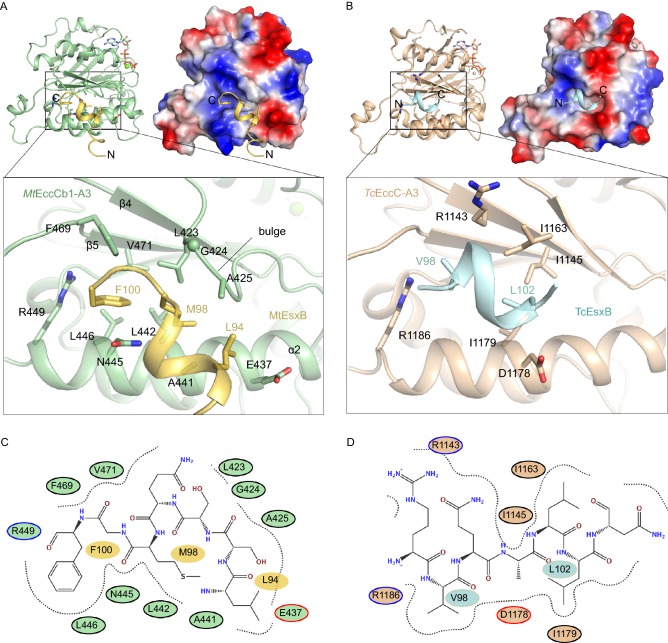
**Substrate-specific recognition of EccCb1-ATPase**_**3**_**from*Mycobacterium tuberculosis*and EccC-ATPase**_**3**_**from*Thermomonospora curvata***. (A) (Upper left) A cartoon image of *Mt*EccCb1-ATPase_3_ (pale green) in complex with *Mt*EsxB (yellow). (Upper right) The same complex but shown as an electrostatic surface. (Bottom image) A zoom-in view of the interactions at binding pocket. N/C, the N-/C-terminal end of peptide. (B) (Upper left) A cartoon image of *Tc*EccC1-ATPase_3_ (wheat) in complex with the C-terminal region of *Tc*EsxB (pale cyan) (PDB code: 4N1A). (Upper right) The same complex but *Tc*EccC1-ATPase_3_ is shown as an electrostatic surface. (Bottom image) A zoom-in view of the interactions at binding pocket. (C) Schematic diagram of the interactions between *Mt*EccCb1-ATPase_3_ and *Mt*EsxB at the recognition pocket. (D) Schematic diagram of the interactions between *Tc*EccC1-ATPase_3_ and *Tc*EsxB at the recognition pocket

Since the structure of the *Thermomonospora curvata* EccCb+EsxB (*Tc*EccCb+*Tc*EsxB) complex (Rosenberg et al. 2015) has been determined, it can be compared with our structure of *Mt*EccCb1-ATPase_3_+*Mt*EsxB complex. In common is that both substrates use their C-terminal sequences for signaling. However, they differ in the composition of the residues that are present. The *Tc*EsxB C-terminal sequence is “^98^VQALLNG^104^” while in *Mt*EsxB it is “^94^LSSQMGF^100^”. Structural superposition showed that the two EsxB proteins bind to the same position on ATPase domains, however the binding pockets are different and accordingly the bound peptides adopt different conformations (Fig. 4A–D). In terms of secondary structure, the C-terminus of *Tc*EsxB forms a helix to participate in binding while *Mt*EsxB also has a helix but its C-terminal end finishes with a short loop. Moreover, the two EsxB substrates bind in opposite orientations.

Detailed analysis of the *Tc*EccCb+*Tc*EsxB structure showed that Val98 and Leu102 of *Tc*EsxB both insert deep into the recognition pocket at positions similar to Phe100 and Met98 in *Mt*EsxB. This despite the fact that the valine and leucine side-chains are comparatively smaller than phenylalanine and methionine. Another difference between the binding modes is that there is no equivalent to Leu94 of *Mt*EsxB in *Tc*EsxB. Comparison of recognition pockets in the two ATPase_3_ structures showed that there is an additional β-strand in *Tc*EccCb-ATPase_3_ instead of the bulge loop near β4 in *Mt*EccCb1-ATPase_3_ (Fig. 4A and 4B). The binding of Phe100 of *Mt*EsxB is through clamping between Asn445 and Phe469 in *Mt*EccCb1-ATPase_3_, however, the corresponding residues are both replaced by alanine in *Tc*EccCb-ATPase_3_ thus clamping cannot occur in *Tc*EsxB. Instead, van der Waals’ forces by residues surrounding Val98 are the main contributors to binding. In terms of buried surface areas, 371 Å^2^ is buried in the interface between *Tc*EccCb-ATPase_3_ and *Tc*EsxB, slightly smaller than the 414 Å^2^ in the *Mt*EccCb1-ATPase_3_+*Mt*EsxB complex. This is in reasonable agreement with the fact that the binding affinities are comparable, ~15 μmol/L for the *Tc*EccCb+*Tc*EsxB (Rosenberg et al. 2015) and 34 μmol/L for the *Mt*EccCb1-ATPase_3_+*Mt*EsxB complex, despite the fact that these affinities were measured by different techniques (fluorescence *vs* ITC). These distinct aspects of substrate binding suggest that T7SSs from different species could use substrate-specific recognition patterns for secretion.

### Comparison of recognition pockets of EccC-ATPase domains

**Figure 5:**
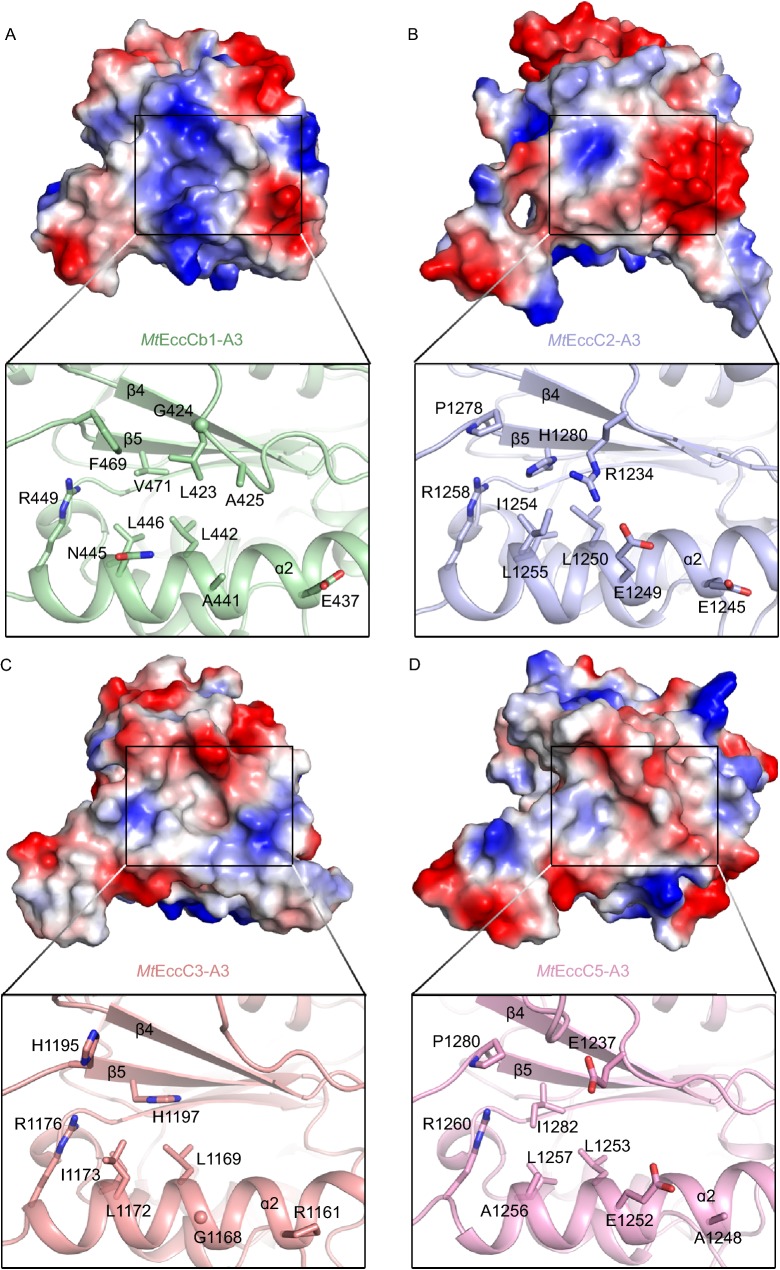
**Structural comparison of the signal recognition pocket of EccC-ATPase**_**3**_**domains**. *Mt*EccCb1-ATPase_3_ (A), *Mt*EccC2-ATPase_3_ (B), *Mt*EccC3-ATPase_3_ (C) and *Mt*EccC5-ATPase_3_ (D) are drawn in electrostatic surface representation. Inserts provide a detailed view of the signal recognition pocket. Comparable to *Mt*EccCb1-ATPase_3_, residues that are likely involved in substrate binding are shown as stick models

A previous report showed that though EsxA and EsxB are absent in supernatants from ΔEsxB and ΔEccD1 deletion *Mtb* strains, the other Esx proteins which may have a signaling module different to “LxxxMxF” are still secreted at the expected levels (Champion et al. 2006). Thus, ESX-2~5 could recognize their own signaling pattern for their substrate Esx proteins even in the absence of EsxA and EsxB. Here, our structural data showed that the recognition pocket in each EccC-ATPase_3_ is different. The signal binding pocket of *Mt*EccC3-ATPase_3_ is much wider than *Mt*EccCb1-ATPase_3_ (Fig. 5A and 5C); the pocket of *Mt*EccC5-ATPase_3_ is much flatter (Fig. 5D) while *Mt*EccC2-ATPase_3_ has a shallow recognition pocket (Fig. 5B). The different shapes and compositions of signal recognition pockets (Figs. 5 and S3) further imply that different T7SS subtypes could specifically recognize individual substrates. In addition, conservation analysis of 143 EccC-ATPase_3_ from different species showed that the signal recognition pocket is hypervariable (Fig. S4B). Interestingly, though ATPase_1_ and ATPase_2_ of *Tc*EccC and *Gt*EssC are homologous with ATPase_3_ of EccC in sequence and structure, their recognition pocket region can interact with the linker peptide connecting two ATPase domains, and auto-inhibit the ATPase activity (Rosenberg et al. 2015; Zoltner et al. 2016). Thus, the functional roles of the recognition pocket of T7SS ATPase domains are in accord with the sequence and structure variation.

## Discussion

Type VII secretion systems are responsible for secreting virulence factors and other effectors (Unnikrishnan et al. 2017). EccC proteins, which typically contain three ATPase domains, have been demonstrated to be required for secretion of substrates by maintaining a stable membrane complex and by acting as a motor to translocate substrates across membrane (Stanley et al. 2003; Houben et al. 2012). In this study, we have provided high-resolution crystal structures of ATPase_3_ domains of EccC proteins from *Mtb*, all in complex with ATP. Like the property of *Tc*EccC and *Gt*EssC (Rosenberg et al. 2015; Zoltner et al. 2016), we proposed that these ATPase_3_ domains are in a state of pre-activation and other components or conformational changes may be needed for their activation.

Negative staining electron microscopy showed the ESX-5 apparatus (including the core subunit EccC) from *Mycobacteriumxenopi* forms a 1.8 MDa transmembrane complex with six-fold symmetry (Beckham et al. 2017). NanoLC-MS/MS analysis of the large T7SS membrane bound complex showed that it contains six copies of EccC (Houben et al. 2012). The EccC is a putative ATPase that belongs to the well-conserved FtsK/SpoIIIE family, of which different members have also been shown to form hexamers (Massey et al. 2006). Thus, a hexameric model for EccCb1-ATPase_3_ can be proposed based on the hexamer structure of FtsK ATPase domain (Massey et al. 2006) through superposition. Indeed, this is similar to what we observed in the packing of our EccC5-ATPase_3_ crystal structure which has six-fold screw axis symmetry (Fig. S8A and S8B). In our hexameric model (Fig. S9A), the ATP binding site is located at the interface between two neighboring ATPase domains. Conformational changes and ATP hydrolysis for the hexamer are required when T7SS is translocating substrate. Similar to FtsK, the protruding segment between ɑ2 and β5 interacts with the loop between β4 and ɑ2 of the neighboring subunit. Such an interaction mode can also be observed in the helical packing of EccC5-ATPase_3_ (Fig. S8C).

The hexameric model of EccCb1-ATPase_3_ forms a central channel with an inner diameter of about 25 Å (Fig. S9A), matching the size of helix bundle of substrates, for example, EsxAB dimer (Fig. S9B) (Renshaw et al. ). The substrate recognition pocket is located at the bottom side, and close to the central pore. The inner surface of the channel is composed of the loop in the V4 region, α6 and the loop between β7 and β8. Note that the loop in the V4 region is at the gating position of the channel and thus may be responsible for the initial entry of substrate. This loop varies in length and residue composition for different ESX systems allowing them to bind their own substrates. Variation of the loop at the gating position and diverse residues inside the channel may also contribute to the substrate selection and translocation for different subtypes of T7SS. The hexameric model could also be proposed for ATPase_1_ and ATPase_2_ domains. We could expect that EccC may use three layers of hexamer structure to drive helix-bundle substrate translocation across membrane (Fig. S9C), this was similar with the proposed hexameric EssC-C model (Zoltner et al. 2016). Considering the flexibility of ATPase domains of EccC5 in the previous reported ESX-5 model, conformational changes are needed to induce hexamer formation.

Loss of the secretion function of T7SS coincides with a reduction in bacterial replication, weakened inflammation, decreasing granuloma formation and an increase in the host’s survival (Hsu et al. 2003; Gao et al. 2004; Guinn et al. 2004; Volkman et al. 2004; Brodin et al. 2006). Considering the fact that the RD1 region, which encodes components of T7SS including EccCb1, is the main distinction between vaccine stain BCG and virulent strains (Mahairas et al. 1996; Behr et al. 1999), the ATPase_3_ domain of EccC, especially *Mt*EccCb1, is therefore a valuable target for developing new anti-TB therapies or vaccines. Based on this study, small molecules or peptides could be designed to bind at the substrate recognition site to block virulence factor secretion. Given the success of secretion of engineered yeast ubiquitin fused to the “LSSQMGF” peptide fragment by ESX-1 (Champion et al. 2006), next generation of vaccine strains could be engineered to secrete sets of immunodominant factors without causing disease, by using the signaling sequence.

## Materials and methods

### Cloning and expression

The DNA coding sequences of *Mt*EccCb1-ATPase_3_ (residues 315-591), *Mt*EccC2-ATPase_3_ (residues 1,127-1,396), *Mt*EccC3-ATPase_3_ (residues 1,060-1,330), *Mt*EccC5-ATPase_3_ (residues 1,125-1,391), as well as *Mt*EsxB alone and *Mt*EsxB-EsxA loci in tandem were PCR amplified from *Mycobacteriumtuberculosis* H37Rv genomic DNA using pairwise primers listed in Table S1. The PCR product of *Mt*EccCb1-ATPase_3_ was cloned into the pET-M3C plasmid, modified from pET-32-M3C (Novagen), fused with N-terminal 6xHis tag and a Rhinovirus 3C protease cleavage site, while constructs of *Mt*EccC3-ATPase_3_ and *Mt*EccC5-ATPase_3_ were made in the pET-22b vector (Novagen) fused with C-terminal 6xHis tag. The sequence of *Mt*EsxB alone, *Mt*EsxB-EsxA tandem sequence and *Mt*EccC2-ATPase_3_ each was cloned into pET-32-M3C (Novagen) vector which expresses a thioredoxin(Trx)-His tag followed by a Rhinovirus 3C protease cleavage site fused at the N-terminus of the protein. Site-directed mutagenesis was performed using the TaKaRa MutanBEST Kit. The mutants were introduced by the PCR method using the EsxB expression plasmid as a template, with pairs of primers encoding the mutations at the sites of substitution. All constructs were verified by sequencing and then transformed into *Escherichia coli* BL21 (DE3) strain for expression. The bacteria were cultured in Luria-Bertani media supplemented with 100 μg/mL ampicillin at 37 °C to an OD_600_ of 0.6. Protein expression was induced by the addition of 0.2 mmol/L IPTG for 20 h at 16 °C. Cells were harvested after centrifugation at 4,000 rpm for 30 min and frozen at −80 °C.

### Protein purification

All ATPase_3_ domain proteins and substrate Esx proteins were purified by a similar method. Briefly, cells were thawed and resuspended in buffer A (20 mmol/L Hepes pH 7.0, 150 mmol/L NaCl, 5% (*w*/*v*) glycerol, 1 mmol/L MgCl_2_, 5 mmol/L ATP). The resuspended cells were then lysed by passing through a French Press at 800 bar after adding 1 mmol/L PMSF. Cell debris was then removed by centrifugation at 18,000 rpm for 30 min at 4 °C. The supernatant was applied to Ni-NTA agarose beads (GE Healthcare) for 2 h at 4 °C. The beads were rinsed with buffer A containing 30 mmol/L imidazole. For *Mt*EccCb1-ATPase_3_, *Mt*EccC2-ATPase_3_ and Esx proteins, the N-terminal tag was cleaved by 3C protease and then eluted. For *Mt*EccC3-ATPase_3_ and *Mt*EccC5-ATPase_3_, the recombinant protein was eluted from the beads with buffer A containing 300 mmol/L imidazole. Then the sample was concentrated and purified using a 5mL Hitrap Q HP (GE life science) column followed by size exclusion chromatography (SEC) using a Superdex 75 HR 10/30 (GE life science) column. The peak fractions were pooled and concentrated to approximately 10 mg/mL using a 10 kDa cut-off spin concentrator (Millipore). The separately purified *Mt*EccCb1-ATPase_3_ and *Mt*EsxB were mixed in a 1:1 molar ratio, incubated and purified again by gel filtration. The fractions containing complex were pooled and concentrated for crystallization.

### Crystallization

Crystallization trials were performed by hanging-drop vapor diffusion method at 16 °C. The protein solution, diluted to 8~10 mg/mL, was mixed in a 1:1 (*v*/*v*) ratio with the reservoir solution. Crystals of *Mt*EccCb1-ATPase_3_, *Mt*EccC2-ATPase_3_, *Mt*EccC3-ATPase_3_, *Mt*EccC5-ATPase_3_ and *Mt*EccCb1-ATPase_3_+*Mt*EsxB were grown from condition I [0.49 mol/L sodium phosphate monobasic monohydrate, 0.91 mol/L potassium phosphate dibasic, pH 6.9], condition II [0.1 M BIS-TRIS pH 6.5, 45% (*v*/*v*) Polypropylene glycol P 400], conditions III [0.1 mol/L Hepes/sodium hydroxide pH 7.5, 0.2 mol/L sodium chloride, 20% (*w*/*v*) PEG3000], condition IV [0.2 mol/L sodium dihydrogen phosphate monohydrate pH 4.5, 20% (*w*/*v*) PEG3350] and condition V [0.1 mol/L MES pH 6.5, 0.2 mol/L sodium chloride, 25% (*w*/*v*) PEG3350], respectively. After optimization for each condition, crystals were harvested using glycerol as cryo-protectant, flash-cooled and stored in liquid nitrogen for data collection.

### Data collection and structure determination

X-ray data were collected on beamlines BL18U1 and BL19U1 at Shanghai Synchrotron Radiation Facility (SSRF) and beamline BL41XU at SPring-8. Data sets were processed, merged and scaled using HKL2000 (Otwinowski and Minor 1997). The initial phases for *Mt*EccCb1-ATPase_3_, *Mt*EccC2-ATPase_3_, *Mt*EccC3-ATPase_3_ and *Mt*EccC5-ATPase_3_ were solved by the molecular replacement method using PHASER (McCoy et al. 2007) using the structure of the ATPase_3_ domain of EccC from *Thermomonospora curvata* (PDB code: 4NH0) (Rosenberg et al. 2015) as the search template. Model building was performed automatically using phenix.autobuild. Manual building in COOT (Emsley and Cowtan 2004) and refinement in PHENIX (Adams et al. 2010) were carried out iteratively for several rounds to obtain the final models. The structure of *Mt*EccCb1-ATPase_3_ in complex with *Mt*EsxB was then solved by molecular replacement using model of *Mt*EccCb1-ATPase_3_ as search template. Data collection and structure refinement statistics are summarized in Table S2.

### Isothermal titration calorimetry (ITC)

ITC was performed at 20 °C with a MicroCal iTC200 instrument (GE Healthcare). Proteins and peptides were prepared in ITC buffer containing 20 mmol/L Hepes (pH 7.0), 150 mmol/L NaCl, 1 mmol/L MgCl_2_, 1 mmol/L ATP and 5% (*w*/*v*) glycerol. The concentration of *Mt*EccCb1-ATPase_3_, Esx proteins and peptides were 50 μmol/L, 1 mmol/L and 1.2 mmol/L, respectively. Control experiments were performed under same experimental conditions except that the sample in the syringe was replaced with the ITC buffer. This allowed the calculation of heat of dilution for the protein. The acquired ITC data were analyzed by the Origin 7.0 (GE Healthcare) program using the “One Set of Binding Sites” fitting model.

### ATPase activity assay

ATPase activities were measured using the ATPase/GTPase Activity Assay Kit (MAK-113, Sigma-Aldrich) according to the manufacturer’s instruction. The purified protein was diluted to 10 μmol/L with Assay Buffer (20 mmol/L Hepes pH 7.0, 150 mmol/L NaCl, 5% (*w*/*v*) glycerol, 5 mmol/L ATP, 5 mmol/L MgCl_2_). 20 μL of the reaction mixture containing the diluted protein was incubated for 1 h at 20 °C. Then, 100 μL of malachite green reagent was added into each reaction well and incubated for 10 min. After that, the absorbance at 620 nm were measured, proportional to the enzyme activity present.

### Data availability

Coordinates and structure factors for *Mt*EccCb1-ATPase_3_, *Mt*EccC2-ATPase_3_, *Mt*EccC3-ATPase_3_, *Mt*EccC5-ATPase_3_ and *Mt*EccCb1-ATPase_3_+*Mt*EsxB complex have been deposited in the Protein Data Bank, under accession codes 6JD4, 6JD5, 6J17, 6J18 and 6J19. All other data are available from the authors upon request.

## Electronic supplementary material

Below is the link to the electronic supplementary material.

Supplementary material 1 (PDF 7518 kb)
