# Supplementary material for: Structural insights into substrate recognition by the type VII secretion system
Source: Protein Cell. 2019 Nov 23;11(2):124–37. doi: 10.1007/s13238-019-00671-z (PMC6954902; doi:10.1007/s13238-019-00671-z)
Supplement: Supplementary file 1 — Supplementary material 1 (PDF 7518 kb) [file 13238_2019_671_MOESM1_ESM.pdf]

Supplementary materials

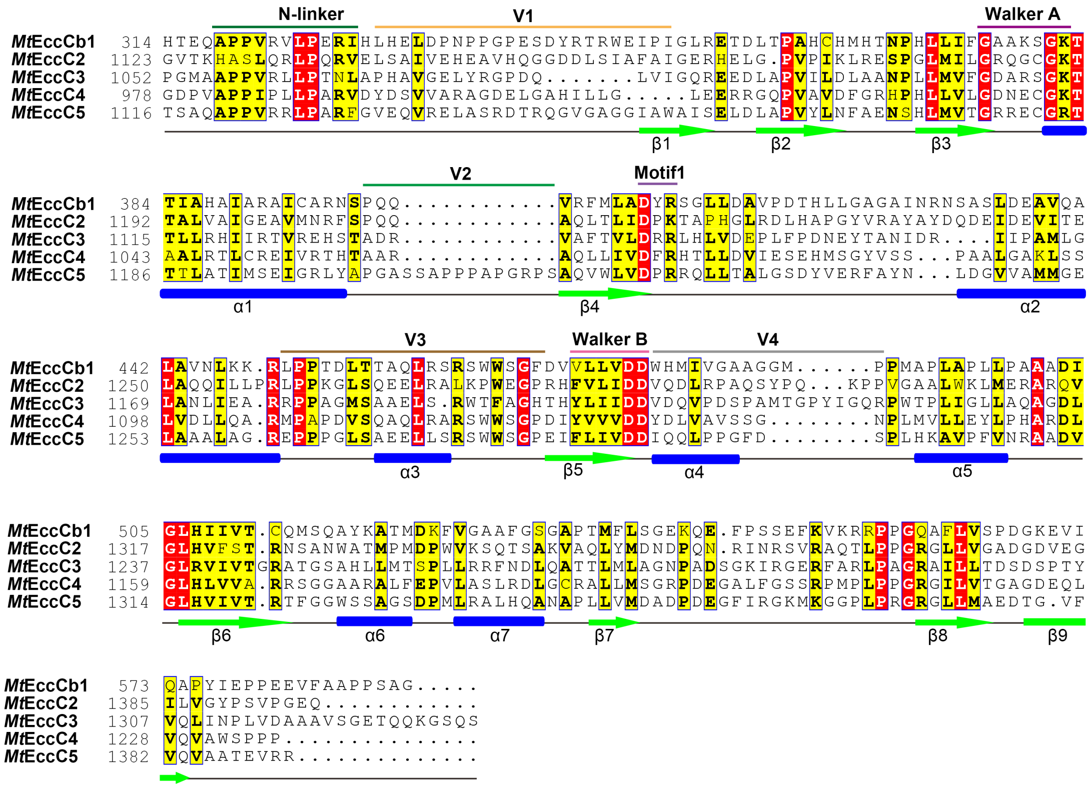

Figure S1. Sequence alignment of EccC-ATPase$_3$ from ESX-1~-5 of *Mycobacterium tuberculosis*.

Secondary structures are labeled below the sequences. Conserved motifs and variable segments are identified above the sequences. V1-4, correspond to variable regions 1-4.

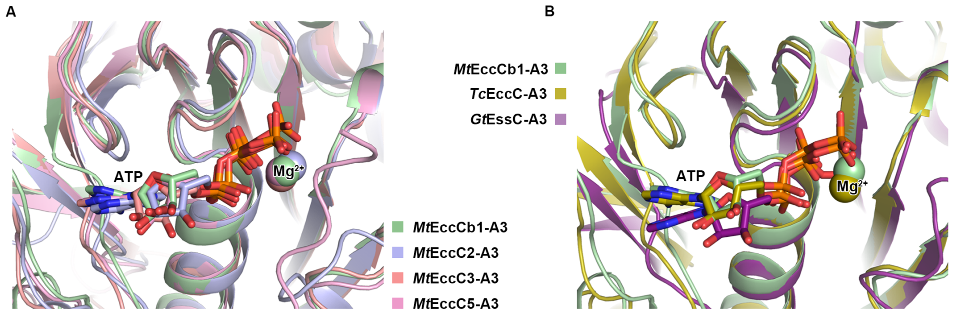

Figure S2. Superposition of *Mt*EccC-ATPase$_3$ structures at the nucleotide-binding site.

(A) The four EccC-ATPase$_3$ structures from *Mycobacterium tuberculosis* are superimposed at the nucleotide-binding site.

(B) EccCb1-ATPase$_3$ from *Mycobacterium tuberculosis*, EccC-ATPase$_3$ from *Thermomonospora curvata* (PDB code: 4NH0) and EssC-ATPase$_3$ from *Geobacillus thermodenitrificans* (PDB code: 5FV0) are superimposed at the nucleotide-binding site.

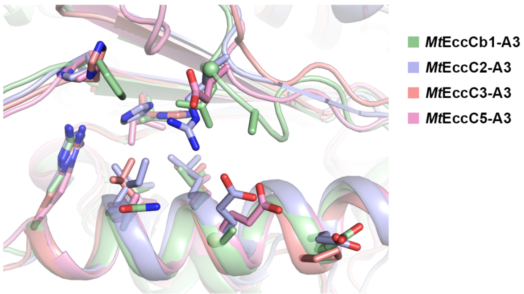

Figure S3. Superposition of the four *Mt*EccC-ATPase$_3$ structures at the signal recognition pocket.

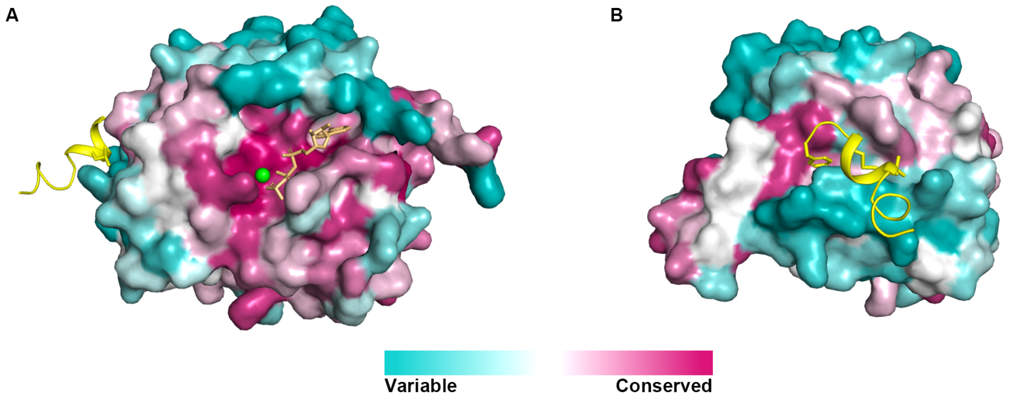

Figure S4. Residues conservation map on *Mt*EccCb1-ATPase$_3$+*Mt*EsxB structure. Residue conservation based on 143 unique EccC sequences was mapped onto the *Mt*EccCb1-ATPase$_3$+*Mt*EsxB structure. The analysis reveals that the ATP (wheat) and magnesium (green) binding sites are highly conserved (A), whereas the signal peptide (yellow) recognition pocket is variable (B).

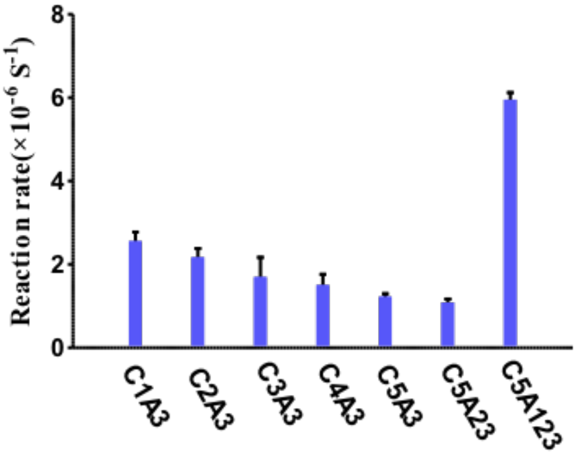

Figure S5. The enzyme activity of ATPase domains of EccC protein from *Mtb*. All the groups exhibit low reaction rate indicating little enzyme activity, even though the EccC5 fragment containing three ATPase domains has a relative higher value than the others. Each bar is the mean and SD of three measurements.

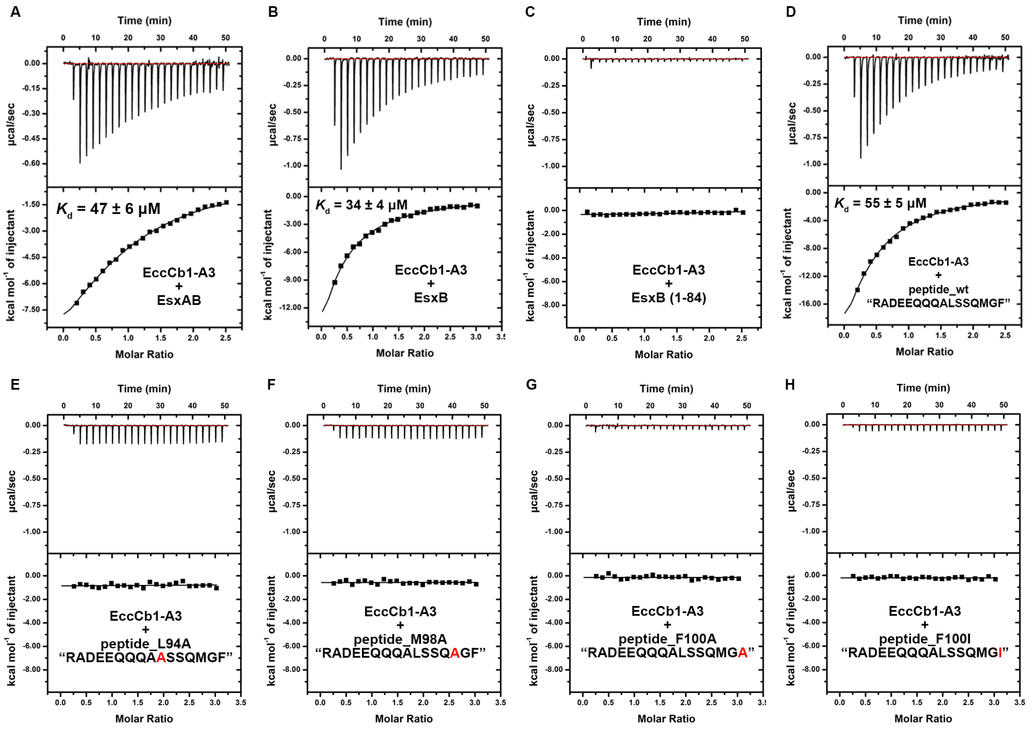

Figure S6. ITC assay analysis for substrate recognition.

The ITC assay shows the binding affinities of (A) EsxAB, (B) EsxB, (C) EsxB (residues 1-84), (D) peptide_wt, (E) peptide_L94A, (F) peptide_M98A, (G) peptide_F100A and (H) peptide_F100I, to EccCb1-ATPase$_3$. The data were representative of at least three repetitions. For E-H, the point mutations are indicated by the red highlight in the sequence.

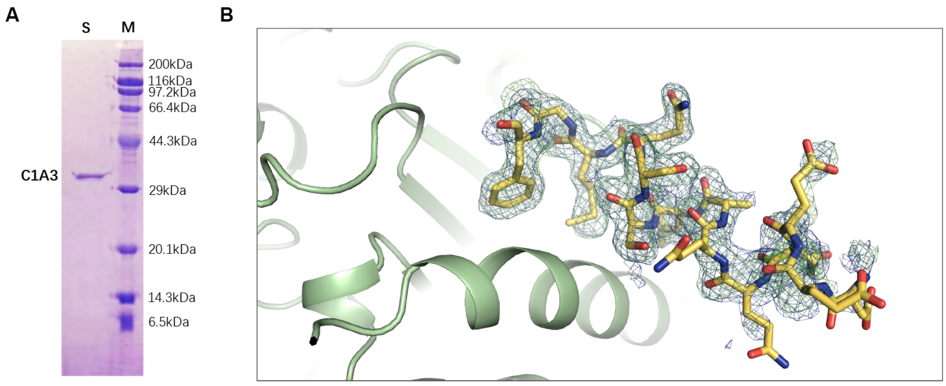

Figure S7. Degradation analysis of *Mt*EccCb1-ATPase$_3$+*Mt*EsxB complex

(A) SDS-PAGE analysis of crystals of *Mt*EccCb1-ATPase$_3$+*Mt*EsxB. *Mt*EccCb1-ATPase$_3$ was stable 4 days after crystallization, while *Mt*EsxB was degraded severely to small pieces that cannot be observed on the gel. S, crystal sample of *Mt*EccCb1-ATPase$_3$+*Mt*EsxB; M, marker.

(B) The omit density of the C-terminal end of *Mt*EsxB in the *Mt*EccCb1-ATPase$_3$+*Mt*EsxB structure. The 2F_o-F_c annealing omit density map (blue mesh) and F_o-F_c annealing omit map (green mesh) are contoured at 1 σ and 3 σ, respectively.

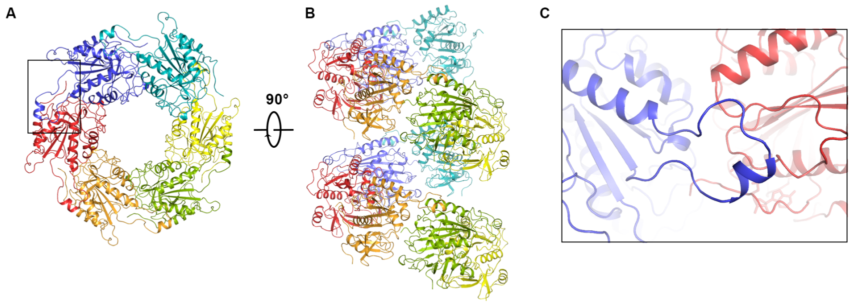

Figure S8. The helical crystal packing of *Mt*EccC5-ATPase$_3$.

(A) The crystal packing of six *Mt*EccC5-ATPase$_3$ looking down the 6-fold screw axis viewed from bottom. The box marks the interaction of the protruding fragment with neighboring molecule.

(B) The helical crystal packing rotated for 90° relative to (A).

(C) Zoom-in view of the protruding fragment interacting with neighboring molecule in the crystal packing.

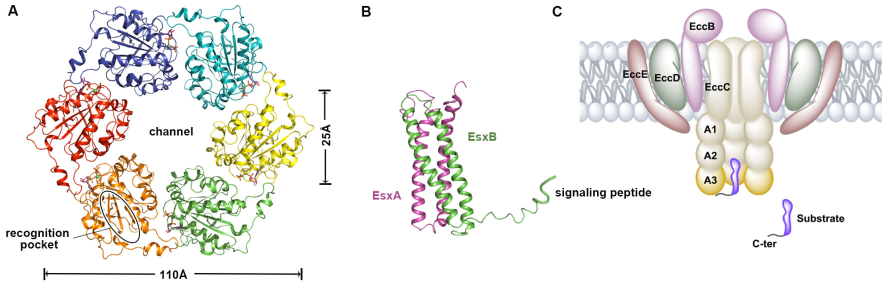

Figure S9. The hexameric model of EccC-ATPase$_3$.

(A) The hexameric model of *Mt*EccCb1-ATPase$_3$ viewed from bottom, with central channel diameter of 25 Å and outer diameter of 110 Å. The location of the substrate recognition pocket is marked with a circle. ATP and Mg^{2+} are shown as a stick model and a sphere, respectively.

(B) The rod-shaped structure of EsxAB complex (PDB code: 1WA8). EsxA and EsxB are colored magenta and green, respectively.

(C) A model of the type VII secretion system translocating substrate protein across membrane. A1/A2/A3, the ATPase$_1$/ATPase$_2$/ATPase$_3$ domain of EccC protein; C-ter, the C-terminal peptide of substrate protein.

Table S1. PCR Primers for constructs.

Constructs	Forward / Reverse Primers (5’->3’)
pEТ-M3C-*Mt*EccCb1<br>(315-591)	F: 5'-CTGTTCCAGGGGCCCGGATCCACCGAACAGGCACCTCCGGTGC-3'<br>R: 5'-GTGGTGGTGGTGGTGGTGCTCGAGTTAACCGGCGCTTGGGGGTGCTGC-3'
pET-32-M3C-*Mt*EccC2<br>(1127-1396)	F: 5'-CCAGGGGCCCGGATCCCACGCAAGTCTGCAGCGGCTGC-3'<br>R: 5'- GTGGTGGTGCTCGAGCTACTGCTCGCCGGGCACCGACG-3'
pET-22b-*Mt*EccC3<br>(1060-1330)	F: 5'-CTTTAAGAAGGAGATATACATATGCGGTTGTTGCCCACCAACCTTGC-3'<br>R: 5'-GTGGTGGTGGTGGTGGTGCTCGAGTCATGACTGACTCCCCTTCTG-3'
pET-22b-*Mt*EccC5<br>(1125-1391)	F: 5'-CTTTAAGAAGGAGATATACATATGCGGTTGCCGGCGCGGTTCGGCG-3'<br>R: 5'-GTGGTGGTGGTGGTGGTGCTCGAGCTACCGACGCACCTCGGTGGC-3'
pET-32-M3C-*Mt*EsxB	F: 5'-TTCTGTTCCAGGGGCCCGGATCCATGGCAGAGATGAAGACCGATGC-3'<br>R: 5'-GGTGGTGGTGGTGGTGCTCGAGTCAGAAGCCCATTTGCGAGGACAG-3'
pET-32-M3C-*Mt*EsxA	F: 5'-GTTTACCAGGGGCCCGGATCCATGACAGAGCAGCAGTGGAATTTCG-3'<br>R: 5'-GGTGGTGGTGGTGCTCGAGCTATGCGAACATCCCAGTGACGTTGC-3'
pET-32-M3C-*Mt*EsxB-<br>*Mt*EsxA	F: 5'-GTTTACCAGGGGCCCGGATCCATGGCAGAGATGAAGACCGATGCC-3'<br>R: 5'-GTGGTGGTGGTGGTGGTGCTCGAGCTATGCGAACATCCCAGTGACGT-3'
pET-32-M3C-*Mt*EsxB<br>(F100A)	F: 5'-GCGTGACTCGAGCACCACCACCACCACCACTGAGATCCG-3'<br>R: 5'-GCTCGAGTCACGCGCCCATTTGCGAGGACAGCGCCTGCTG-3'

Table S2. Data collection and refinement statistics.

	*Mt*EccCb1-ATPase$_3$	*Mt*EccC2-ATPase$_3$	*Mt*EccC3-ATPase$_3$	*Mt*EccC5-ATPase$_3$	*Mt*EccCb1-ATPase$_3$+*Mt*EsxB
**Data collection**					
Space group	$P2_12_12_1$	$P2_1$	$P2_12_12_1$	$P6_5$	$C222_1$
Cell dimensions					
a, b, c (Å)	61.22, 77.31, 106.01	51.45, 69.69, 75.96	53.36, 56.63, 93.53	89.16, 89.16, 62.64	81.58, 129.65, 64.20
α, β, γ (°)	90, 90, 90	90, 101, 90	90, 90, 90	90, 90, 120	90, 90, 90
Resolution (Å)	50.00-2.10 (2.21-2.10)[a]	50.00-2.20 (2.32-2.20)	50.00-1.97 (2.00-1.97)	50.00-2.00 (2.03-2.00)	50.00-1.98 (2.01-1.98)
Unique reflections	30,107	26,929	19,248	19,280	23,760
Completeness (%)	100.0 (100.0)	99.9 (100.0)	93.2 (67.3)	100.0 (100.0)	98.0 (77.5)
R_{merge}	0.134 (0.331)	0.091 (0.642)	0.116 (0.448)	0.123 (0.440)	0.189 (0.674)
Redundancy	12.9 (12.9)	6.9 (7.0)	5.5 (3.7)	4.9 (4.6)	10.5 (5.5)
$I/\delta(I)$	3.6 (1.8)	7.3 (1.2)	13.4 (1.7)	16.5 (2.8)	13.0 (1.1)
$CC_{1/2}$	0.998 (0.988)	0.999 (0.886)	0.999 (0.777)	0.999 (0.841)	0.999 (0.728)
Wilson B factors (Å^2)	16.4	33.4	30.1	27.5	28.1
**Refinement**					
Resolution (Å)	43.72-2.10	38.71-2.20	46.35-1.98	38.61-2.00	45.61-1.98
No. of Reflections	29,963	26,880	19,172	19,257	23,744
R_{work} / R_{free} (%)	20.2 / 24.6	17.6 / 22.5	18.1 / 22.8	16.7 / 20.0	17.0 / 20.8
No. of non-H atoms					
Protein	3986	4037	1955	2087	2105
Ligand/ion	64	64	32	32	32
Water	386	216	148	214	221
Average B factor (Å^2)					

	*Mt*EccCb1-ATPase$_3$	*Mt*EccC2-ATPase$_3$	*Mt*EccC3-ATPase$_3$	*Mt*EccC5-ATPase$_3$	*Mt*EccCb1-ATPase$_3$+*Mt*EsxB
Protein	22.23	40.51	44.68	34.26	33.38
Ligand/ion	12.76	34.70	25.94	24.85	26.94
Water	27.01	40.35	44.38	39.88	42.24
R.m.s deviations					
Bond lengths (Å)	0.003	0.007	0.015	0.003	0.007
Bond angles (°)	0.715	0.976	1.328	0.810	0.857
Ramachandran plot (%)					
Favored	97.5	97.3	96.8	97.8	97.4
Allowed	2.5	2.7	3.2	2.2	2.6
Outliers	0.0	0.0	0.0	0.0	0.0

[a] The values for the highest shell are shown in parentheses.

Table S3 The *r.m.s.d.* values between different ATPase$_3$ domains after superposition.

r.m.s.d. (Å) / Cα pairs	C1A3	C2A3	C3A3	C5A3
C1A3		1.27	1.05	0.99
C2A3	152		2.02	1.30
C3A3	140	162		1.22
C5A3	151	171	154	
